# Sleep Deprivation Related Changes of Plasma Oxytocin in Males and Female Contraceptive Users Depend on Sex and Correlate Differentially With Anxiety and Pain Hypersensitivity

**DOI:** 10.3389/fnbeh.2018.00161

**Published:** 2018-08-02

**Authors:** Sigrid Schuh-Hofer, Nicole Eichhorn, Valery Grinevich, Rolf-Detlef Treede

**Affiliations:** ^1^Department of Neurophysiology, Centre of Biomedicine and Medical Technology Mannheim, Medical Faculty Mannheim, Heidelberg University, Mannheim, Germany; ^2^Clinic for Neurology, Medical Faculty Mannheim, Heidelberg University, Mannheim, Germany; ^3^Schaller Research Group on Neuropeptides, German Cancer Research Center, Heidelberg and Central Institute of Mental Health, Mannheim, Germany

**Keywords:** sleep deprivation, oxytocin, pain, anxiety, sex, descending pain modulation, quantitative sensory testing

## Abstract

Disturbed sleep is known to substantially aggravate both the pain condition and the affective state of pain patients. The neurobiological mechanisms underlying these adverse effects are unknown. Oxytocin (OT), being largely involved in social and emotional behavior, is considered to also play a modulatory role in nociception. We hypothesized a pathophysiological role of OT for the hyperalgesic and anxiogenic effects of sleep loss. An established human model of one night of total sleep deprivation (TSD) was used to test this hypothesis. Twenty young healthy students (*n* = 10 male and *n* = 10 female) were investigated in a balanced cross-over design, contrasting TSD with a night of habitual sleep (HS). All females took monophasic oral contraceptives (OC) and were investigated during their ‘pill-free’ phase. Plasma OT concentrations were correlated with (1) pain thresholds, (2) descending pain inhibition, and (3) state-anxiety scores. Compared to the HS condition, the plasma OT concentration was significantly increased in sleep deprived females (*p* = 0.02) but not males (*p* = 0.69). TSD resulted in pain hypersensitivity to noxious cold (*p* = 0.05), noxious heat (*p* = 0.023), and pricking stimuli (*p* = 0.013) and significantly increased state-anxiety (*p* = 0.021). While, independent of sex, lower heat pain thresholds correlated with higher plasma OT (*p* = 0.036), no such associations were found for cold/mechanical pain. In sleep-deprived females, higher plasma OT showed a mild (but insignificant) association with lower pain inhibition (*p* = 0.093). We found a positive correlation between anxiety-scores and OT (*p* = 0.021), which was enhanced when respecting “sex” (*p* = 0.008) and “sleep” (*p* = 0.001) in a hierarchical regression analysis. Altogether, our study revealed a complex and partially sex-dependent correlation between plasma OT and TSD-induced changes of experimental pain and anxiety. The minor role of OT for TSD-induced changes of evoked pain, and its major involvement in anxiety, argues against a specific role of OT for linking the adverse effects of TSD on pain sensitivity and anxiety with each other. Future investigations are needed in order to dissect out the effect of OC on the sex-dependent effects of TSD observed in our study.

## Introduction

Disturbed sleep belongs to the major complaints of pain patients (Taylor et al., 2007; Finan et al., 2013; Nijs et al., 2018). Clinical and experimental studies have shown that sleep deprivation is able to substantially aggravate the pain condition of pain patients (Kundermann et al., 2004a; Smith and Haythornthwaite, 2004; Sivertsen et al., 2015). Even more, sleep disturbances and pain are considered to be mutually reinforcing, thus leading to a vicious cycle (Benca et al., 2004; Sanford, 2008). Though being clinically highly relevant, the neurobiological mechanisms underlying the pro-nociceptive effects of disturbed sleep are barely known. Beyond its impact on pain, disturbed sleep is also known to deteriorate the affective state of pain patients. Namely, the anxiogenic effect of disturbed sleep is well acknowledged (Sagaspe et al., 2006; Babson et al., 2010; Pires et al., 2016; Goldstein-Piekarski et al., 2018). Only recently, the anxiogenic effect of sleep deprivation has been shown to be correlated with brain morphology changes in the ventromedial prefrontal cortex and – in women – with reduced gray matter volume in the anterior insula and lateral orbitofrontal cortex (Goldstein-Piekarski et al., 2018). Part of these cortical areas – like the insula and the ventromedial prefrontal cortex – do not only belong to the affective-motivational dimension of pain processing (Peyron et al., 2000; Apkarian et al., 2011; Mutschler et al., 2012; Kragel et al., 2018) but are also target regions of the neurohypophysial hormone oxytocin (OT) (Ross and Young, 2009; Riem et al., 2011). This neuropeptide, being largely recognized for its role in social behavior (Neumann, 2008; Macdonald and Macdonald, 2010; Striepens et al., 2011) and its modulatory effects on anxiety (MacDonald and Feifel, 2014; Neumann and Slattery, 2016), has recently gained considerable attention with respect to its involvement in nociceptive processing (Gonzalez-Hernandez et al., 2014; Boll et al., 2017; Gamal-Eltrabily and Manzano-Garcia, 2017; Xin et al., 2017). Several kinds of mechanisms have been described by which OT may contribute to the modulation of nociceptive processing. Supraspinally, extrahypothalamic projections of OT neurons to limbic brain regions, like the anterior cingulate cortex and amygdala, suggest a role of OT for the affective dimension of pain processing (Knobloch et al., 2012; Grinevich et al., 2016). At the brainstem level, anatomical (Campbell et al., 2009; Lee et al., 2013) and functional (Yang et al., 2011) data indicate that OT is involved in descending pain modulation of the PAG-RVM (periaqueductal gray – rostral ventromedial medulla) pathway, where it interacts with the opioidergic system. Via hypothalamo-spinal projections to dorsal horn neurons, OT is involved in nociceptive processing at the spinal cord level (Schoenen et al., 1985; Condés-Lara et al., 2015). Only recently, a peripheral mode of action was proposed according to which OT may interact with nociceptive-specific nerve terminals in the skin (Gonzalez-Hernandez et al., 2017).

During the last decade, the anti-nociceptive potency of OT has been intensively studied. Surprisingly, while preclinical evidence strongly points to a considerable role of OT in pain-related behavior, results from human studies are highly equivocal. Like recently summarized, only about half of the previous human studies on the involvement of OT in nociception were able to confirm anti-nociceptive effects of OT, while the remaining studies failed to convincingly demonstrate a pain modulating role. Differences between methodological approaches to explore the role of OT (exogenous application versus assessment of endogenous levels) and between outcome measures (spontaneous pain versus evoked pain) may particularly contribute to the large heterogeneity of study results [see (Rash et al., 2014; Tracy et al., 2015; Boll et al., 2017; Xin et al., 2017) for review].

Compared to the field of pain research, little is known about the contribution of the OT-system for the physiology and pathophysiology of sleep. Only chronobiological aspects have been addressed, indicating that the OT-system is not subject to circadian variations (Amico et al., 1983; Blagrove et al., 2012). We aimed to explore the neurobiological correlates underlying sleep deprivation induced changes of pain sensitivity and state anxiety. Backed by current knowledge, we hypothesized a pathophysiological role of OT for both the deterioration of pain and the affective state of sleep deprived individuals. To address this topic, a well-established human experimental model of one-night total sleep deprivation (TSD) was used. This model has been shown to increase the sensitivity to noxious stimuli (Kundermann et al., 2004b; Haack et al., 2009; Schuh-Hofer et al., 2013; Eichhorn et al., 2017) [see (Schrimpf et al., 2015) for review] and to be anxiogenic (Schuh-Hofer et al., 2013; Short and Louca, 2015; Eichhorn et al., 2017). We hypothesized (1) a significant impact of sleep deprivation on the activity of the OT-system and, hence, on OT-release into the blood circulation and (2) a significant correlation between the concentration of plasma OT and TSD-induced alterations of evoked pain and anxiety. Since the involvement of OT in social and anxiety-related behavior is highly gender-specific (Macdonald, 2012; Dumais et al., 2013; Rilling et al., 2014; Scheele et al., 2014; Tracy et al., 2015; Luo et al., 2017; Bredewold and Veenema, 2018), the role of sex was explicitly respected in our study. Therefore, a highly homogenous sex-mixed study population (*n* = 10 females and *n* = 10 males) was investigated. All females took monophasic oral contraceptives OC (see details below) and were strictly investigated during their “pill-free” phase. The effect of TSD on the nociceptive system was assessed by (1) using a QST battery (Rolke et al., 2006) and (2) a “Cold Pressor Test,” which enables to estimate the individual’s capacity to inhibit pain (Yarnitsky, 2010; Yarnitsky et al., 2015). The impact of TSD on anxiety was evaluated by using the State-Anxiety Inventory (Spielberger et al., 1970). Finally, the salivary concentration of cortisol as a flanking biomarker for the stress-level of our study subjects was determined.

## Materials and Methods

This study was carried out in accordance with the Declaration of Helsinki and in accordance with the recommendations of the Local Ethical Committee of the Medical Faculty of Mannheim. The protocol was approved by the Local Ethical Committee of the Medical Faculty of Mannheim. All subjects gave written informed consent prior to study enrollment in accordance with the Declaration of Helsinki.

### Subjects

The study was performed in a subset of the previously described study population of *n* = 36 study participants (Eichhorn et al., 2017) and comprised ten young healthy female (age 24.0 ± 2.7, mean ± SD; range 22–30 years) and male (23.4 ± 1.8; range 22–26 years) students. Somatic or psychiatric diseases and, in particular, pain disorders or insomnia were excluded in all study participants by taking their medical history, performing a physical and neurological examination and by using psychological questionnaires BDI (Beck et al., 1961), STAI-Trait Inventory (Spielberger et al., 1970). Both BDI-scores (females: 0.2 ± 0.7 and males: 1.0 ± 1.5, mean ± SD) and Trait Anxiety Scores (females: 28.1 ± 5.1 and males: 27.6 ± 4.2, mean ± SD) were within normal limits and did not indicate depression or anxiety. Primary pain or headache disorders were excluded as well as any serious pain-associated physical trauma. The ICHD-II classification criteria (Headache Classification Subcommittee of the International Headache Society, 2004) were used to explicitly exclude patients with a diagnosis of migraine [1.1, 1.2] or probable migraine [1.6]. Importantly, females complaining of a peri-menstrual syndrome were excluded from the study. Sleep histories were taken from all study participants and the PSQI (Buysse et al., 1989) (range: 0–21; scores < 5: good sleepers without clinical signs of insomnia) was used to quantitatively assess their individual sleep quality. According to our results (PSQI: females: 1.4 ± 1.1 and males: 1.8 ± 1.1, mean ± SD), none of the study participants suffered from sleep disturbances. A lark and owl questionnaire (Wilson, 1988) was used to determine sleep preferences and to exclude extreme morning (score ≥ 70) or extreme evening types (score ≤ 30). Results from this questionnaire indicated that all but two study participants belonged to the “neutral type” (females: 51.4 ± 3.7 and males: 50.3 ± 6.3, range 43–59) with two males ranging at the border to the “moderate evening type” (Score: 59). Demographic data are shown in **Table 1**.

**Table 1:** Demographic data.

No.	BDI	STAI-trait	PSQI	Lark/Owl
1	0	22	1	54
2	0	26	1	55
3	0	27	0	51
4	0	35	3	43
5	0	22	1	55
6	0	37	3	49
7	2	28	1	53
8	0	28	1	51
9	0	28	1	52
10	2	32	1	51
11	0	27	3	52
12	2	30	1	50
13	2	25	1	59
14	4	26	4	50
15	0	28	2	42
16	0	21	1	46
17	0	36	1	44
18	1	30	1	44
19	0	25	2	59
20	0	33	4	52


*BDI, beck depression inventory (range 0–20); STAI-Trait, trait scores of the state and trait anxiety inventory (range 20–80); PSQI, Pittsburgh sleep quality index (range 0–21); Lark/Owl, Lark/Owl questionnaire (range 16–86; 16–30: extreme evening type; 31–41: moderately evening type, 42–58: neutral type; 59–69: moderately morning type; 70–86: extreme morning type).*

None of the study participants took analgesics. All study participants were non-smokers. They were asked to abstain from caffeine or any caffeinated beverages at least three days before the experiment took place. Since estrogens are known to impact on OT synthesis and release (Patisaul et al., 2003; Windle et al., 2006; Choleris et al., 2008; Macdonald, 2012), it was important to study all female subjects in the same phase of their menstrual cycle and – since contraceptives are also known to impact on OT (Silber et al., 1987; Chiodera et al., 1991; Salonia et al., 2005; Scheele et al., 2016) – to use a homogenous female study population with respect to contraceptives. This condition was fulfilled by the fact that all female study participants were on monophasic contraceptives. In nine of ten females, the concentration of ethinylestradiol was 0.03 mg per tablet, while the contraceptive pill of one female contained 0.02 mg ethinylestradiol per tablet. All females were exclusively studied during their “pill pause” (day 3 to 7). In seven of the ten female subjects, the experiments were performed at exactly the same day of their menstrual cycle.

### Study Protocol

All study subjects filled in sleep diaries at least five days before experiments took place. To objectively monitor their sleep-wake cycles, participants were asked to carry a commercially available battery-operated piezoelectric device (Actiwatch^®^ Device, Philips Respironics, Amsterdam) around the wrist. Continuous motion data, recorded by this Actiwatch Device, are translated into periods of ‘activity’ and periods of ‘rest’ by using the Respironics Actiwatch Software (Respironics Actiware^®^ Version 5.59.0015) [see (Schuh-Hofer et al., 2013) for details]. We used a balanced cross-over design with the same number of participants (*n* = 5 for each gender), either starting with a night of habitual sleep (HS) or a night of TSD. The time-interval between the two study experiments was adjusted to the female cycle. To avoid biasing due to alternation of the sleep-wake rhythm from weekend to weekday, experiments never took place on Mondays.

For the condition “HS,” study participants were allowed to spend the night at home to avoid artificial surroundings which may have influenced their sleep quality. The night of TSD was spent at our Institute, where a medical student played games and chatted with the study participants to keep him/her continuously active. For double-check, participants had to keep wearing the Actiwatch device also during the sleep deprivation night.

Analysis of Actiwatch data revealed an average total sleep time on weekdays of 7.3 ± 0.4 and 8.8 ± 0.6 h at weekends. As expected, the total sleep time at the sleep deprivation night was 0 ± 0 h.

### Study Schedule

Experiments started at eight o’clock in the morning. After a drug screening test (Drug-Screen, nal von minden GmbH, Germany) and before serving breakfast, saliva (cortisol) and blood samples (OT) were collected. All participants ate a standard breakfast (white bread, butter, honey, fruit, or herbal tea). Thereafter, subjects had to fill in the State-Anxiety Inventory of the STAI. As a next step, QST was performed on the non-dominant hand to determine the effect of TSD on nociception (duration: 30 min). Upon completion of QST, a cold pressor test was performed (see below).

### Outcome Measures

#### Quantitative Sensory Testing (QST)

To explore the effect of TSD on pain sensitivity, a QST procedure, developed by the German Research Network on Neuropathic Pain (DFNS), was used. A detailed description on testing procedures and quantitative assessment has been formerly published (Schuh-Hofer et al., 2013; Eichhorn et al., 2017). The following description is restricted to the assessment of nociceptive thresholds, since the presented study only relates to these parameters. We used the TSA II NeuroSensoryAnalyzer (MEDOC, Israel) to assess the effect of TSD on thermal pain thresholds. Temperature changes were run in ramps of 1°C/s with a low cut-off of 0°C and a high cut-off of 50°C. Starting from a baseline-temperature of 32°C, the temperature of the thermode (surface: 9 cm^2^) was decreased/increased three times and the subjects were asked to press a PC mouse button as soon as they perceived a change from the impression of a warm/cold sensation toward an additional impression of a “burning,” “stinging,” “drilling,” or “aching” sensation. The arithmetical mean of three measurements was calculated to determine CPT and HPT. For measuring MPTs of pricking forces, pinprick devices with a flat contact area of 0.25 mm diameter, ranging from 8 to 512 mN (MRC systems, Heidelberg, Germany), were used. Pinprick devices were applied in five series of ascending and descending stimulus intensities. MPT was then determined by calculating the geometric mean of the 5 series of ascending and descending stimulus intensities. A pressure algometer (Wagner Instruments, Greenwich-CT, probe diameter 1.0 cm^2^, up to 2000 kPa pressure) was applied on the thenar eminence with a pressure ramp of 50 kPa/s. The arithmetical mean of three measurements was then calculated to determine the PPT.

For details of the instruction, see http://www.neuro.med.tu-muenchen.de/dfns/pdfs/QST-Investigators_brochure_Version_2_1_final_english_2010_07_09.pdf.

#### Assessment of Descending Pain Inhibition: “Cold Pressor Test”

To determine the individual’s capacity to inhibit pain, the impact of ice cold water – serving as conditioning stimulus – on PPTs was assessed (Yarnitsky, 2010; Yarnitsky et al., 2015). PPT was determined by applying the same pressure algometer already used for QST (see above). Study participants were instructed to immerse their dominant hand up to ∼12 cm of the distal forearm into a tank filled with ice-cold water with their fingers spread apart. The temperature (0–2°C) was measured by a calibrated mercury thermometer (0–100°C). Subjects were instructed to pull their hand out of the water as soon as the painful sensation became intolerable with a predetermined maximum exposition time of 180 s. The impact of the conditioning stimulus (ice cold water) on the test stimulus (PPT) was assessed by subtracting PPT_post_ from PPT_prae_ (=PPT_prae-post_).

To correlate plasma OT levels with the individual endogenous capacity to inhibit pain, a ratio between PPT_prae-post_ (TSD) and PPT_prae-post_ (HS) was calculated (=Ratio PPT_prae-post_). By doing so, a value >1.0 indicated that the conditioning stimulus (ice cold water) resulted in an increased capacity to inhibit pain, while values <1.0 indicated a change toward pain facilitation.

#### Blood/Saliva Sampling and Hormone Analysis

##### Blood samples

We used 5 ml of EDTA blood for the assessment of plasma OT concentrations. After venous puncture, the 5 ml EDTA vacutainer tubes were immediately cooled in ice-chilled water at 4°C. The samples were then centrifuged at 4°C at 4000 rpm for 5 min, aliquoted and stored at -20°C. After completion of the study, plasma samples were sent on dry ice to Munich, Germany. After extraction using LiChroprep Si60 (Merck), the samples were analyzed using a sensitive radioimmunoassay (RIAgnosis, Munich, Germany; see (Martin et al., 2014). The assay detection limit was 0.1 pg/sample, and cross-reactivity with other related neuropeptides was <0.7%. The coefficient of variation for intra-assay precision was <8%, whereas inter-assay variation was eliminated by measuring all samples within the same assay.

##### Saliva

Saliva was collected by using cortisol salivettes with a synthetic swab (Reg. Nr. 51.1534.500, Sarstedt Company, Nümbrecht, Germany). The saliva samples were stored at -20°C until shipping to the Biochemical Laboratory, Trier University, Germany, where the samples were analyzed. Saliva samples were centrifuged at 2000 *g* for 10 min. 100 ul of saliva were used for duplicate analysis. Cortisol levels were determined employing a competitive solid phase time-resolved fluorescence immunoassay with flouromeric end point detection (DELFIA). Standards, controls (saliva pools) and samples were given in duplicate wells. Cortisol concentrations of the samples were calculated with VICTOR X4^TM^ Multilabel Plate Reader (Perkin Elmer, MA, United States). The intra-assay coefficient of variation was between 4.0 and 6.7%. Inter-assay variation was eliminated by measuring all samples within the same assay [see (Dressendörfer et al., 1992)].

### Statistical Analysis

We used RM-ANOVA analyses [within subject’s factor: “sleep” (HS or TSD), between subject’s factor: sex] to determine the impact of sleep deprivation on the outcome parameters. Prior to statistical analysis, the QST parameters PPT and MPT had to be transformed logarithmically in order to achieve normal distribution (Rolke et al., 2006). For direct comparison of outcome parameters under the two conditions HS and TSD, paired *t*-tests were additionally performed.

To determine the overall relation between OT levels and the respective outcome parameters, linear regression analyses were performed. Thereafter, a hierarchical model of analysis was applied to specifically evaluate the contribution of “sex” and of “sleep” (HS versus TSD) on our results. Therefore, the respective dependent variable of interest was first entered to the model and “OT” was determined as an independent variable (step 1). Thereafter, “sex” (step 2) and “sleep” (step 3) were added to the model.

Statistics were done using the software package IBM SPSS Statistics 20. The significance level was set to α ≤ 0.05. Cohen’s d and partial eta squared were used as the measure for effect size.

All data passed tests for normality (Normality test Shapiro–Wilk) and homogeneity of variance. Mauchley’s Test of Sphericity, applied in RM-ANOVA analyses, was in no case violated.

## Results

### Effect of TSD on OT

**FIGURE 1:**
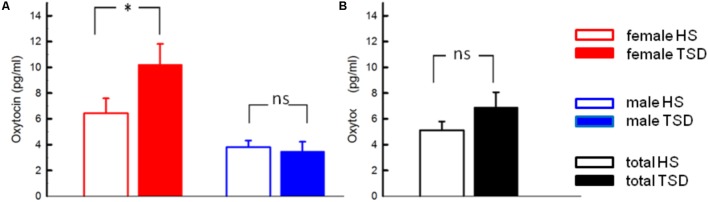
Effect of sleep deprivation on plasma oxytocin and cortisol. **(A)** We found a significant effect of TSD on oxytocin levels in females (left), but not males (right). **(B)** Based on the entire study population, TSD just failed to change oxytocin levels (*p* = 0.057). ^∗^*p* < 0.05; data are given as mean ± SEM.

According to RM-ANOVA, there was a significant main effect for OT regarding sex (*p* = 0.002) and a significant interaction between the sleep condition (HS versus TSD) and sex (*p* = 0.026). “Sleep” condition as a main factor just failed to have a significant effect on OT levels (*p* = 0.057). Separate analyses on the effect of TSD on OT-levels in females and males revealed a 58.9 ± 40.5% increase in females (*p* = 0.02), while plasma OT levels of males remained unchanged or even tended to decrease (8.7 ± 16.6 decrease; *p* = 0.34) (see **Figure 1** and **Table 2**).

**Table 2:** RM-ANOVA.

	Sleep			Sex			Sleep ^∗^ sex		
			
	*p*	*F*	$\eta_{p}^{2}$	*p*	*F*	$\eta_{p}^{2}$	*p*	*F*	$\eta_{p}^{2}$
Oxytocin	0.057	4.134	0.187	0.002	13.164	0.422	0.026	5.874	0.246
Cortisol	0.241	1.470	0.075	**0.048**	4.508	0.200	0.273	1.279	0.066
CPT	**0.050**	3.470	0.162	0.763	0.094	0.005	0.584	0.311	0.017
HPT	**0.023**	6.221	0.257	0.257	1.368	0.071	0.036	5.155	0.223
Log MPT	**0.013**	7.564	0.296	0.646	0.219	0.012	0.040	4.896	0.214
Log PPT	0.119	2.676	0.129	**0.020**	6.524	0.266	0.748	0.106	0.006
PPT_prae-post_	0.739	0.115	0.006	0.812	0.058	0.003	0.013	7.533	0.295
State anxiety	**<0.001**	23.984	0.571	0.740	0.113	0.006	0.429	0.653	0.035


*RM-ANOVA: within subjects factor: sleep (HS vs. TSD), between subjects factor: sex $\eta_{p}^{2}$: partial eta squared. Sleep ^∗^sex: interaction between Sleep and sex. Values marked in bold indicate significancy of results.*

### Effect of TSD on Cortisol

We found a main effect of sex on saliva cortisol levels (*p* = 0.048, *F* = 4.508; **Table 2**). Neither the sleep condition (*p* = 0.241; *F* = 1.470) nor the interaction between sleep and sex had a significant impact on cortisol levels (*p* = 0.273; *F* = 1.279; see **Table 2**).

### Effect of TSD on Pain Parameters and Anxiety

**FIGURE 2:**
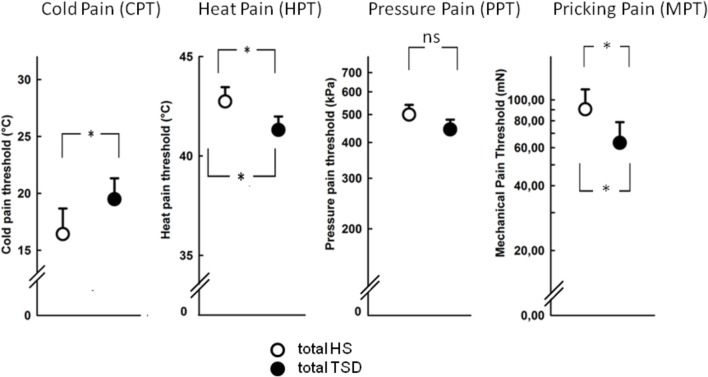
Effect of sleep deprivation on nociceptive thresholds. The figures depict raw data of quantitative sensory testing, based on the mixed gender population. There was a main effect of the sleep condition on thermal pain sensitivity (CPT, HPT). With respect to mechanical submodalities, the sensitivity to pricking stimuli (MPT), but not to blunt pressure (PPT), was significantly increased. ^∗^*p* < 0.05; data are given as mean ± SEM.

**FIGURE 3:**
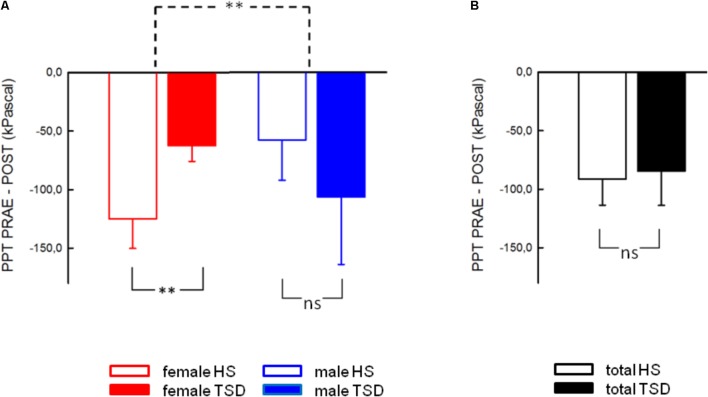
Effect of sleep deprivation on descending pain inhibition. **(A)** The females’ capacity to inhibit pain was significantly decreased after TSD, while males tended to exhibit stronger pain inhibition. **(B)** Consequently, the effect of TSD on the entire study population was not significant. Solid line brackets below vertical bars indicate results from paired *t*-tests, while the dashed bracket in **(A)** relates to the significant sleep ^∗^ sex interaction (RM-ANOVA). ^∗∗^*p* < 0.01; data are given as mean ± SEM.

The mandatory prerequisite of our study was to induce pain hypersensitivity and increased state anxiety levels by TSD. In accordance with this goal, study subjects developed cold hyperalgesia (CPT, *p* = 0.05), heat hyperalgesia (HPT, *p* = 0.02) and hyperalgesia to pricking pain (logMPT, *p* = 0.013) after TSD, while PPTs were not significantly affected (see **Figure 2** and the column “sleep” of **Table 2**). **Table 3** shows that the effect sizes related to this study population are similar to those of our previously published larger study group, of which our study participants represent a subgroup [see (Eichhorn et al., 2017)]. We found a significant sex ^∗^ sleep condition interaction with respect to HPT and logMPT, with females becoming more sensitive to heat (*p* = 0.036) and males becoming more sensitive to pricking pain (*p* = 0.040) (see **Table 2** and **Figure 2**). Like previously shown (Eichhorn et al., 2017), the effect of TSD on descending pain inhibition, represented by the parameter “PPT_prae-post_,” did strongly depend on sex (sex ^∗^ sleep interaction: *p* = 0.013). In females, descending pain inhibition was strongly impaired after TSD (*p* = 0.008). On the contrary, the capacity to inhibit pain tended to be even stronger after TSD in the male study population (*p* = 0.21). Results are depicted in **Figure 3** and RM-ANOVA analysis is given in **Table 2**.

**Table 3:** Effect sizes.

	Cohen’s *d*	*p*	Cohen’s *d*	*p*
	*n* = 20	*n* = 20	*n* = 36	*n* = 36
CPT	-0.469	0.050	-0.731	<0.001
HPT	0.505	0.023	0.626	<0.001
logPPT	0.374	0.11	0.343	0.049
logMPT	0.560	0.013	0.541	0.002
STAI-State	-1.052	<0.001	-0.937	<0.001
PPT_prae-post_ total	-0.065	0.739	-0.021	0.670
PPT_prae-post_ female	-0.760	0.008	-0.381	0.023
PPT_prae-post_ male	0.301	0.210	0.115	0.480


*The columns two and three depict effect sizes and *p*-values for TSD-induced changes of outcome parameters in our study population of *n* = 20 study participants. Data from columns four and five refer to the recently published larger data set of *n* = 36 study participants (Eichhorn et al., 2017). Apart from logPPT, which failed to be significant in our smaller data set (*p* = 0.11); results are comparable with each other. CPT, cold pain threshold; HPT, heat pain threshold; PPT, pressure pain threshold; MPT, mechanical pain threshold; STAI-State, state anxiety inventory.*

#### State Anxiety

**FIGURE 4:**
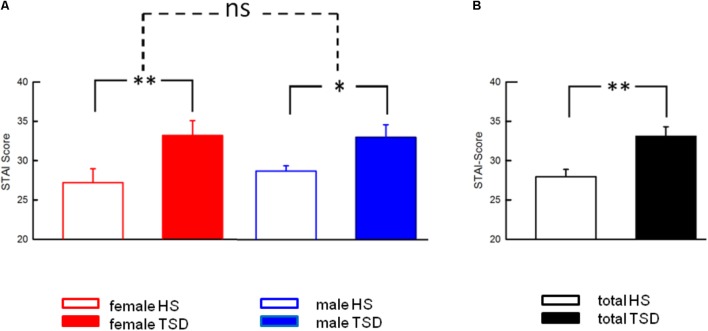
Effect of sleep deprivation on anxiety levels. **(A)** The significant effect of TSD on State Anxiety was prominent in both females and males, and consequently in the entire study population **(B)**. Accordingly, there was no significant sleep ^∗^ sex interaction. Solid line brackets are related to paired *t*-tests. Dashed line brackets relate to the sex ^∗^ sleep interaction (RM-ANOVA). ^∗∗^*p* < 0.01; ^∗^*p* < 0.05; data are given as mean ± SEM.

Anxiety levels, assessed by the STAI-State Inventory, increased significantly after sleep deprivation (HS: 28.0 ± 0.9; TSD: 33.1 ± 1.2; *p* = < 0.001). This effect was independent of gender (RM-ANOVA: sleep ^∗^ sex interaction, *p* = 0.429; see **Table 2**). **Figure 4** depicts results both separated by sex and based on the mixed gender population.

### Correlation Analyses and Hierarchical Regression Analyses

#### Correlation Between OT and Nocicepive Thresholds

**FIGURE 5:**
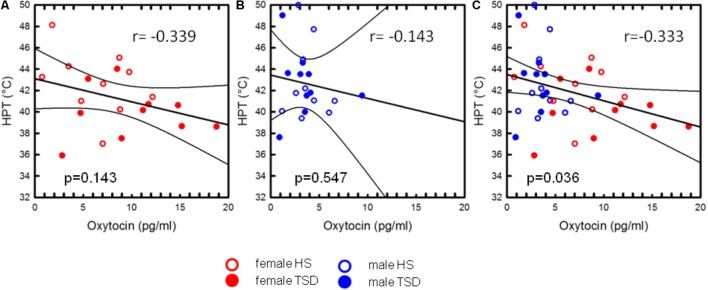
Correlation between OT and HPT. The mild association between heat pain thresholds and plasma-OT concentrations was only significant on the basis of the sex-mixed study population [*p* = 0.036, **(C)**], but not when separately analysing data from females **(A)** or males **(B)**. The variable “sex” did not significantly account for the correlation between HPT and OT-levels.

Oxytocin-levels neither correlated with CPTs nor with Pressure Pain or MPTs (see **Table 4**). However, based on the entire study population, we found a significant correlation between plasma OT-levels and HPTs. Higher OT levels were associated with a stronger sensitivity to noxious heat (*r* = -0.333; *r*^2^ = 0.111; *p* = 0.036, **Figure 5C** and **Table 4**). When separating results by sex, bivariate correlations were not significant (females: *p* = 0.143 and males: *p* = 0.547; see **Figures 5A,B** and **Table 4**). Accordingly, neither adding the variable ‘sex’ (0.3% increase of explained variance) nor ‘sleep’ (3.1% increase of explained variance) to a model of hierarchical regression analysis resulted in reinforcement of the result, thus indicating that none of these two variables is of further predictive value (**Table 5A**).

**Table 4:** Correlations between OT levels and outcome parameters.

	Mixed sex			Female			Male		
			
	*R*	*p*	*F*	*R*	*p*	*F*	*r*	*p*	*F*
CPT	0.007	0.965	0.002	0.128	0.592	0.298	0.067	0.777	0.083
HPT	-0.333	**0.036**	4.748	-0.339	0.143	2.342	-0.143	0.547	0.376
PPT	0.243	0.130	2.392	0.023	0.924	0.009	0.027	0.925	0.009
MPT	0.236	0.142	2.245	0.375	0.104	2.938	0.143	0.548	0.376
Ratio PPT_prae-post_	0.186	0.251	1.359	0.386	**0.093**	3.149	0.340	0.143	2.347
STAI	0.363	**0.021**	5.766	0.650	**0.002**	13.135	0.087	0.717	0.136
Cortisol	0.031	**0.897**	0.017	0.821	**0.004**	16.487	0.385	0.271	0.149


*This table depicts the correlation coefficient (*r*) between the respective outcome parameter and the oxytocin concentration of study participants. Results are given for both the mixed gender population and for the female and male subpopulation. CPT, cold pain threshold; HPT, heat pain threshold; PPT, pressure pain threshold; MPT, mechanical pain threshold; MPS, mechanical pain sensitivity. The ratio PPT_*post/prae*_ enables to estimate whether the exposition to ice cold water resulted in pain inhibition (value > 1.0) or facilitation (value < 1.0). STAI, State Anxiety Score of the STAI-Inventory. Values marked in bold indicate significancy of results.*

**Table 5A:** Hierarchical Regression Model: Correlation between HPT and Oxytocin.

HPT	Full model				Sex			Sleep			Oxy		
				
	*F*	*p*	*r*	*r^2^*	*B*	*T*	Sig	*B*	*T*	Sig	*B*	*t*	Sig
Oxy	4.744	0.036^b^	0.333^a^	0.111							-0.333	-2.178	-0.036
Oxy sex	2.383	0.106^c^	0.338^b^	0.114	0.067	0.361	0.720				-0.296	-1.597	0.119
Oxy sex sleep	2.031	0.127^d^	0.380^c^	0.145	0.096	0.515	0.610	-0.180	-1.135	0.264	-0.244	-1.279	0.209


*Oxytocin and HPT are significantly correlated (*p* = 0.036). However, the predictive power is only marginally increased when adding “sex” (0.3% increase) or “sleep” (3.1%) to the model.*

#### Correlation Between OT and Descending Pain Inhibition

**FIGURE 6:**
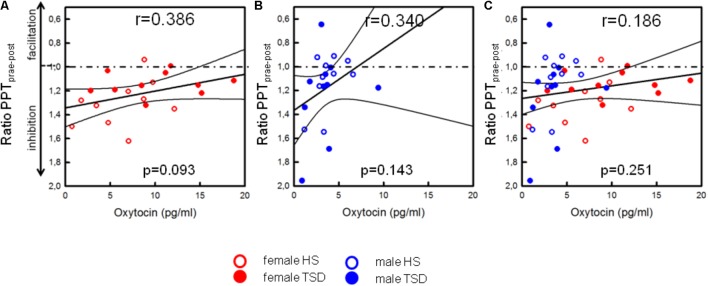
Correlation between OT and descending pain inhibition. Separated by sex, plasma OT-levels of females [**(A)**, *p* = 0.093], but not males **(B)**, tended to be higher in those subjects who had a lower capacity to inhibit pain [*p* = 0.093, **(A)**]. Values below the dashed line indicate pain inhibition while values above the line indicate pain facilitation due to ice cold water exposition. Based on the sex-mixed population, results were not significant **(C)**.

On the basis of the mixed sex population, the capacity to inhibit pain was not correlated with OT-levels (*p* = 0.251, see **Table 4** and **Figure 6C**). Contrary to males (see **Figure 6B** and **Table 4**), there was a mild tendency of less pain inhibition being associated with higher OT levels (*r* = 0.386, *p* = 0.093; **Figure 6A** and **Table 4**).

#### Correlation Between OT and STAI

**FIGURE 7:**
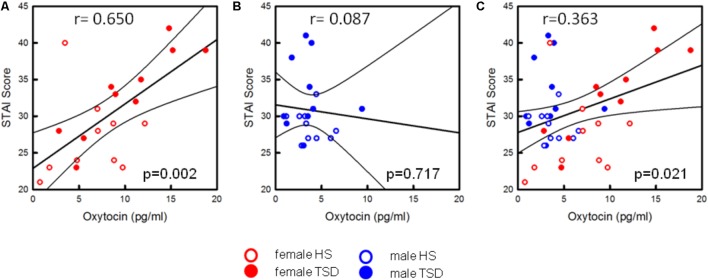
Correlation between OT-levels and state anxiety. Only the state anxiety scores of females are correlated with increased OT-levels [*p* = 0.002, see **(A)**]. In males, the anxiogenic effect of TSD does not seem to be related to the oxytocin level (*p* = 0.717, see **B**]. Based on the entire study population **(C)**, results are still significant (*p* = 0.021).

Based on the sex-mixed population, the correlation between anxiety levels and OT concentrations was significant (*r* = 0.363; *r*^2^ = 0.132; *p* = 0.021; **Figure 7C**). When separating results by sex, a strong association between OT concentrations and STAI-scores was only found in females (*r* = 0.650; *p* = 0.002; **Figure 7A**), not males (*r* = 0.087; *p* = 0.717, **Figure 7B**). Conducting a hierarchical regression analysis, the predictive power of OT (∼13.2% of explained variance) was further increased when adding the variables “sex” (*r*^2^ = 0.229; *p* = 0.008) and “sleep” (*r*^2^ = 0.371; *p* = 0.001) to the model (see **Table 5B**).

**Table 5B:** Hierarchical Regression Model: correlation between STAI and oxytocin.

STAI	Full model				Sex			Sleep			Oxy		
				
	*F*	*p*	*r*	*r^2^*	*B*	*t*	Sig	*B*	*T*	Sig	*B*	*t*	Sig
Oxy	5.766	0.021	0.363	0.132							0.363	2.401	0.021
Oxy sex	5.508	0.008	0.479	0.229	0.375	2.166	0.037				0.569	3.292	0.002
Oxy sex sleep	7.084	0.001	0.609	0.371	0.312	1.951	0.059	0.388	2.849	0.007	0.456	2.791	0.008


*Oxytocin accounts for 13.2% of variability regarding STAI-scores (13.2%. *p* = 0.021). This significant result is further increased to *p* = 0.008 by adding “sex” to the model. Entering the variable “sleep” to the model explains another 14.2% of variance. Since at this point the significance level of the variable “sex” decreases to 0.059, the overlapping effect of “sex” and “sleep” seems to be stronger carried by “sleep” than “sex.”*

### Correlation Between Cortisol and OT

Based on the entire study population, OT and cortisol were not significantly correlated with each other (*p* = 0.734, see **Table 5C**). Only when entering the variable “sex” to the hierarchical regression analysis, the predictive power is significantly increased (full model: *p* = 0.027). Adding the ‘sleep condition’ does only explain another 1.0% of variance and reduces the significance just below the significance level (*p* = 0.056).

**Table 5C:** Hierarchical Regression Model: correlation between salivary cortisol and oxytocin.

Cortisol	Full model				Sex			Sleep			Oxy		
				
	*F*	*p*	*r*	*R^2^*	*B*	*T*	Sig	*B*	*T*	Sig	*B*	*t*	Sig
Oxy	0.117	0.734	0.055	0.003							0.055	0.342	0.734
Oxy sex	3.977	0.027	0.421	0.177	-0.500	-2.796	0.008				-0.220	-1.232	0.226
Oxy sex sleep	2.760	0.056	0.432	0.187	-0.483	-2.657	0.012	-0.103	-0.668	0.508	-0.190	-1.023	0.313


*While absolute values of cortisol and oxytocin do not correlate with each other, entering the variable “sex” to the model is able to increase the predictive power by 17.4%. Adding the variable “sleep” to the model does only explain another 1.0% of variance in the full model. This finding indicates that our finding is mainly carried by sex.*

## Discussion

### Effect of TSD on Plasma OT Levels

We found a sex-dependent effect of TSD on the plasma OT-concentration of study participants. To our knowledge, human studies on the contribution of OT to the pathophysiology of sleep disruption are lacking so far. Only two rodent studies have been published on this topic. Results of these studies, however, were contradictory. Fujihara and co-authors (Fujihara et al., 2003) were unable to demonstrate significant changes of OT mRNA-levels in hypothalamic nuclei of *male* rats when using a short-term (6 h) paradigm of selective REM-sleep deprivation. On the contrary, Pardo et al. (2016) observed an increase of plasma OT-levels in sleep restricted *pregnant rats*, who underwent a long-term paradigm of cumulative sleep restriction (20 h/daily) over six days. Study inconsistencies may not only be explained by methodological differences (regarding, for example, the sleep restriction model or the choice of outcome parameters) but also by differences regarding sex and the hormonal state of the study animals. In our human study, sex revealed to have a crucial impact on study results since plasma levels of OT did only increase in sleep deprived females, not males. However, due to the fact that all female study participants were taking monophasic contraceptives, our findings may not hold true for naturally cycling women. In addition to the fact that *endogenous* estrogens are capable to profoundly activate OT synthesis and release (Pfaff et al., 2011; Acevedo-Rodriguez et al., 2015), exogenous estrogens (derived from OC) have been shown to increase plasma OT-levels (Silber et al., 1987; Chiodera et al., 1991; Salonia et al., 2005). Based on this knowledge, the intake of ethinylestradiol may have had a significant impact on our study results. In order to evaluate the particular contribution of OC to sleep deprivation effects, future sleep deprivation studies thus need to specifically compare naturally cycling females with females taking OC.

### Correlation Between Plasma OT Levels and Pain Thresholds

Using correlation analyses, our study failed to show a significant association between plasma OT concentrations and cold pain, pricking, or pressure pain. Regarding cold pain, our negative finding is in accordance with Grewen and co-authors (Grewen et al., 2008), who were hardly able to show an association between plasma OT and the tolerance to noxious cold when respecting the factor ‘ethnicity’ in their analysis. In contrast to our methodological approach, two other studies used exogenously applied OT to explore its effect on cold pain. Results, however, were contradictory. While Zunhammer and co-authors (Zunhammer et al., 2015) were unable to demonstrate a significant effect of intranasal OT on CPTs, Rash and Campbell (2014) reported a significant increase of CPTs and a decrease of perceived cold pain intensity/unpleasantness after administration of OT. Altogether, at least these studies on the correlation between *endogenous* OT and evoked cold pain failed to support the hypothesis of plasma OT being predictive for cold pain sensitivity in humans.

In contrast to cold pain, we found a significant – but sex and sleep-independent – correlation between plasma OT levels and HPTs. This finding indicates that – contrary to what we expected – *sleep deprivation* does not play a decisive role in the correlation between plasma OT-levels and HPTs. The previous investigations on the impact of OT on heat pain were highly equivocal. Both attenuation (Zunhammer et al., 2015; Paloyelis et al., 2016) and enhancement of perceived heat pain intensity (Tracy et al., 2017) have been reported, but a lack of effect has also been observed (Grewen et al., 2008; Kessner et al., 2013; Eisenach et al., 2015; Zunhammer et al., 2016). Compared to psychophysical studies, Paloyelis and co-authors used laser-heat evoked potentials (LEP) to explore the effect of intranasal OT on heat pain in healthy males. They found that OT was not only able to attenuate subjective pain ratings but to also reduce the amplitudes of the N1 and the N2-component of LEP’s. LEP’s represent an objective measure of nociceptive processing and reflect the cortical response to noxious radiant heat. Thus, in some agreement with our own finding, the LEP-study provides with some objective electrophysiological evidence for an involvement of OT in heat pain perception.

We failed to show an association between the perception of noxious mechanical stimuli and plasma OT-levels. The previous studies on a modulatory role of the OT-system for pricking and pressure pain are lacking. In fact, except for thermal pain perception, knowledge on the effect of OT on other nociceptive modalities like visceral pressure pain (Louvel et al., 1996) or electrical pain (Singer et al., 2008) relies on single study observations. Thus, we are currently unable to seriously discuss the role of OT for mechanical pain perception in this study. Furthermore, studies, which address the impact of OT on the entire spectrum of the nociceptive system, are needed to advance our understanding of the contribution of OT for the perception of different nociceptive modalities.

### Descending Pain Inhibition

In accordance with our previously published results on sex-dependent effects of TSD on descending pain inhibition, only sleep-deprived female OC-users showed impairment of their endogenous pain control (Eichhorn et al., 2017). Accordingly, we found a sex-dependent (but insignificant) correlation between pain inhibition and OT. While, in females, higher plasma OT-concentrations showed – by tendency – an association with lower capacities to inhibit pain, a similar effect in males was lacking.

So far, only one previous study focused on the impact of OT on endogenous pain control. In this study, intranasally applied OT had a significant effect on descending pain control. This effect, however, was dependent on the site at which the outcome parameter was measured. While OT did not modulate conditioned pressure pain measured at the forearm, enhanced pain inhibition was observed at the masseter muscle (Goodin et al., 2015). Though the authors argue that the route of OT administration (intranasally) may have resulted in a preferred effect of the neuropeptide on the trigeminally innervated anatomical site, the site-specific aspect of this study nevertheless remains elusive. Unfortunately, apart from the study performed by Goodin et al., human studies on the correlation between OT and the endogenous capacity to inhibit pain are currently missing. Thus, in view of our currently sparse and heterogeneous data, we strongly support to continue human research on the modulatory effect of OT on supraspinal pain control in humans.

### OT and Anxiety

Contrary to its minor contribution to TSD-induced effects on experimental pain, OT had a considerable predictive power regarding state anxiety. This finding is in line with a large body of evidence for an important role of the OT-system for anxiety and fear (Heinrichs et al., 2003; MacDonald and Feifel, 2014; Eckstein et al., 2015; Grund et al., 2017). Notably, sex revealed to play an important role in our study. Similar sex-dependent effects have been observed by Weisman and co-authors, who explored the correlation between Trait Anxiety-scores and plasma OT-concentrations. According to their finding, males showed an inverse correlation between plasma OT-levels and Trait Anxiety scores, while a subgroup of females with particular high anxiety scores exhibited the reverse – i.e., a positive correlation (Weisman et al., 2013). Opposing findings have also been observed in other neurophysiological domains of anxiety. In males, higher plasma OT-levels correlated with lower “attachment anxiety,” while the reverse was observed in females (Marazziti et al., 2006; Weisman et al., 2013). Unfortunately, none of these studies controlled for the use of OC. Thus, we are not able to further discuss putative differences between naturally cycling females and OC-users. An OC-independent role of female sex has, however, been shown in a study from our team, where we demonstrated that OT plays a key role in attenuation of anxiety during lactation – a physiological state known to drastically boost the OT-system (Menon et al., 2018). Altogether, in light of our currently limited knowledge, it becomes obvious that future studies urgently need to take the putative impact of OC’s into account. By contrasting naturally cycling females with female OC-users and with males, we will be able to more specifically determine the probable sex-dependent role of OT for modulating anxiety.

### OT and Cortisol

Irrespective of the sleep condition, females showed higher basal cortisol levels than males. This may be due to hormonal bias by OC since – independent of the menstrual cycle – elevated plasma cortisol levels have been observed in females taking OC when compared to those using natural contraception methods (Kirschbaum et al., 1999; McQuaid et al., 2016).

According to our findings, cortisol levels of sleep deprived study participants did not significantly increase. Animal experiments on the cortisol response to sleep loss indicate that activation of the HPA-axis depends on the duration and the model of sleep deprivation (Meerlo et al., 2002; Fujihara et al., 2003; Wodarski et al., 2015). Accordingly, our short-term protocol of sleep deprivation may, on average, have been only a mild stressor – at least from the perspective of young healthy medical students. Since OT is discussed to be dynamically interrelated with cortisol (Heinrichs et al., 2003; Cardoso et al., 2014; Brown et al., 2016), we finally aimed to assess the association between these two stress-related biomarkers at the individual level. Based on the sex-mixed study population, we were not able to show a significant correlation between OT and cortisol. However, “sex” revealed to play a significant role since the predictive power of OT increased markedly when respecting ‘sex’ in a hierarchical regression analysis. In the literature, OT is discussed to buffer the cortisol response to stress. Results, however, have been equivocal and neither sex differences nor the impact of OC have been specifically addressed so far (Cardoso et al., 2014; Brown et al., 2016; McQuaid et al., 2016). Therefore, we strongly suggest performing the future studies in order to explore the impact of endogenous and exogenous sex hormones on the putative cortisol buffering effect of OT.

#### Methodological Considerations

We applied rigid inclusion/exclusion criteria and used highly standardized procedures to minimize confounding factors. By double-monitoring of study participants during the sleep deprivation night, via an Actiwatch Device and a student assistant, TSD was ensured. Apart from the fact that, inherent to the study design, the significance of our data may not be extrapolated to naturally cycling women, several drawbacks of the study need to be discussed.

(1) An important methodological limitation of our study relates to the fact that our model is not able to induce spontaneous pain. Thus, our analyses had to be restricted to study correlations between plasma OT and evoked pain. Another drawback relates to exclusively focusing on “anxiety” as an affective outcome measure. Contrary to the previous studies (Goodin et al., 2015; Zunhammer et al., 2015; Zunhammer et al., 2016), our study did not include other parameters like the “Profile of Mood States” (POMS) or an “Unpleasantness-scale” to further evaluate the role of OT regarding the affective domain. Consequently, apart from “anxiety,” we are not able to further comment on the impact of OT on the emotional aspects of pain in our sleep deprivation model.
(2) Our study did not integrate a night of “restorative sleep” in order to show the reversibility of our finding. Future studies, thus should extend the study protocol and include repetition of experiments under the condition ‘restorative sleep’. This approach will help to further strengthen the significance of the presented study findings.
(3) Finally, we assessed OT in the blood, but not in the cerebrospinal fluid. Although this approach is common (Alfven et al., 1994; Alfven, 2004; Grewen et al., 2008), uncertainty remains whether plasma OT can be regarded an adequate outcome parameter for studying causal relations between OT effects and specific behavioral patterns (Kagerbauer et al., 2013). However, although the cerebrospinal fluid space would have been the preferred target compartment in our study, ethical reasons prevented us from collecting CSF in healthy study participants.

## Conclusion

Our findings suggest that sleep-deprivation related changes of OT are not causally linked to TSD-induced thermal and mechanical hyperalgesia. In addition, OT may only play a minor role for impaired descending pain inhibition of sleep deprived females. In contrast to its neglectable contribution to altered pain thresholds, OT appears to be particularly involved in females’ increased anxiety-levels. This discrepancy suggests that TSD induced effects on anxious mood and experimental pain are – at least with respect to the biomarker OT – differentially regulated. Notably, the significance of our data relates to females using OC’s. In addition, our study population was investigated under artificial experimental conditions. We strongly suggest re-investigating the role of OT in a patient cohort since our experimental findings still do not exclude that OT may contribute to the perceived intensity of spontaneous pain in chronic pain patients suffering from sleep disturbances.

## Author Contributions

SS-H and R-DT designed the study. NE carried out the experiments. SS-H and NE performed the data analysis. SS-H, R-DT, and VG evaluated the data interpretation. SS-H and VG wrote the manuscript. All authors discussed results and commented on the manuscript.

## Conflict of Interest Statement

The authors declare that the research was conducted in the absence of any commercial or financial relationships that could be construed as a potential conflict of interest.

## Abbreviations

BDI: beck depression inventory
CDT: cold detection threshold
CPM: conditioned pain modulation
CPT: cold pain threshold
DMA: dynamic mechanical allodynia
HPT: heat pain threshold
ICHD: international headache classification
MDT: mechanical detection threshold
MPT: mechanical pain threshold
OT: oxytocin
PPT: pressure pain threshold
PSQI: Pittsburgh sleep quality index
QST: quantitative sensory testing
SD: standard deviation
SEM: standard error of the mean
STAI: state and trait anxiety inventory
TSD: total sleep deprivation

## References

[B1] Acevedo-Rodriguez A. Mani S. K. Handa R. J. (2015). Oxytocin and estrogen receptor beta in the brain: an overview. *Front. Endocrinol.* 6:160. 10.3389/fendo.2015.00160 26528239 PMC4606117

[B2] Alfven G. (2004). Plasma oxytocin in children with recurrent abdominal pain. *J. Pediatr. Gastroenterol. Nutr.* 38 513–517. 10.1097/00005176-200405000-00010 15097440

[B3] Alfven G. de la Torre B. Uvnas-Moberg K. (1994). Depressed concentrations of oxytocin and cortisol in children with recurrent abdominal pain of non-organic origin. *Acta Paediatr.* 83 1076–1080. 10.1111/j.1651-2227.1994.tb12989.x 7841708

[B4] Amico J. A. Tenicela R. Johnston J. Robinson A. G. (1983). A time-dependent peak of oxytocin exists in cerebrospinal fluid but not in plasma of humans. *J. Clin. Endocrinol. Metab.* 57 947–951. 10.1210/jcem-57-5-947 6619269

[B5] Apkarian V. A. Hashmi J. A. Baliki M. N. (2011). Pain and the brain: specificity and plasticity of the brain in clinical chronic pain. *Pain* 152 S49–S64. 10.1016/j.pain.2010.11.010 21146929 PMC3045648

[B6] Babson K. A. Trainor C. D. Feldner M. T. Blumenthal H. (2010). A test of the effects of acute sleep deprivation on general and specific self-reported anxiety and depressive symptoms: an experimental extension. *J. Behav. Ther. Exp. Psychiatry* 41 297–303. 10.1016/j.jbtep.2010.02.008 20231014 PMC2862829

[B7] Beck A. T. Ward C. H. Mendelson M. Mock J. Erbaugh J. (1961). An inventory for measuring depression. *Arch. Gen. Psychiatry* 4 561–571. 10.1001/archpsyc.1961.01710120031004 13688369

[B8] Benca R. M. Ancoli-Israel S. Moldofsky H. (2004). Special considerations in insomnia diagnosis and management: depressed, elderly, and chronic pain populations. *J. Clin. Psychiatry* 65(Suppl. 8), 26–35. 15153065

[B9] Blagrove M. Fouquet N. C. Baird A. L. Pace-Schott E. F. Davies A. C. Neuschaffer J. L. (2012). Association of salivary-assessed oxytocin and cortisol levels with time of night and sleep stage. *J. Neural Transm.* 119 1223–1232. 10.1007/s00702-012-0880-1 22911329

[B10] Boll S. Almeida de Minas A. C. Raftogianni A. Herpertz S. C. Grinevich V. (2017). Oxytocin and pain perception: from animal models to human research. *Neuroscience* 10.1016/j.neuroscience.2017.09.041 [Epub ahead of print]. 28965836

[B11] Bredewold R. Veenema A. H. (2018). Sex differences in the regulation of social and anxiety-related behaviors: insights from vasopressin and oxytocin brain systems. *Curr. Opin. Neurobiol.* 49 132–140. 10.1016/j.conb.2018.02.011 29518698 PMC6055524

[B12] Brown C. A. Cardoso C. Ellenbogen M. A. (2016). A meta-analytic review of the correlation between peripheral oxytocin and cortisol concentrations. *Front. Neuroendocrinol.* 43 19–27. 10.1016/j.yfrne.2016.11.001 27836673

[B13] Buysse D. J. Reynolds C. F. III Monk T. H. Berman S. R. Kupfer D. J. (1989). The pittsburgh sleep quality index: a new instrument for psychiatric practice and research. *Psychiatry Res.* 28 193–213. 10.1016/0165-1781(89)90047-4 2748771

[B14] Campbell P. Ophir A. G. Phelps S. M. (2009). Central vasopressin and oxytocin receptor distributions in two species of singing mice. *J. Comp. Neurol.* 516 321–333. 10.1002/cne.22116 19637308

[B15] Cardoso C. Kingdon D. Ellenbogen M. A. (2014). A meta-analytic review of the impact of intranasal oxytocin administration on cortisol concentrations during laboratory tasks: moderation by method and mental health. *Psychoneuroendocrinology* 49 161–170. 10.1016/j.psyneuen.2014.07.014 25086828

[B16] Chiodera P. Volpi R. Capretti L. Marchesi C. d’Amato L. De Ferri A. (1991). Effect of estrogen or insulin-induced hypoglycemia on plasma oxytocin levels in bulimia and anorexia nervosa. *Metabolism* 40 1226–1230. 10.1016/0026-0495(91)90220-Q 1943752

[B17] Choleris E. Devidze N. Kavaliers M. Pfaff D. W. (2008). Steroidal/neuropeptide interactions in hypothalamus and amygdala related to social anxiety. *Prog. Brain Res.* 170 291–303. 10.1016/s0079-6123(08)00424-x 18655890

[B18] Condés-Lara M. Martínez-Lorenzana G. Rubio-Beltrán E. Rodríguez-Jiménez J. Rojas-Piloni G. González-Hernández A. (2015). Hypothalamic paraventricular nucleus stimulation enhances c-Fos expression in spinal and supraspinal structures related to pain modulation. *Neurosci. Res.* 98 59–63. 10.1016/j.neures.2015.04.004 25933550

[B19] Dressendörfer R. A. Kirschbaum C. Rohde W. Stahl F. Strasburger C. J. (1992). Synthesis of a cortisol-biotin conjugate and evaluation as a tracer in an immunoassay for salivary cortisol measurement. *J. Steroid Biochem. Mol. Biol.* 43 683–692. 10.1016/0960-0760(92)90294-S 1472460

[B20] Dumais K. M. Bredewold R. Mayer T. E. Veenema A. H. (2013). Sex differences in oxytocin receptor binding in forebrain regions: correlations with social interest in brain region- and sex- specific ways. *Horm. Behav.* 64 693–701. 10.1016/j.yhbeh.2013.08.012 24055336

[B21] Eckstein M. Becker B. Scheele D. Scholz C. Preckel K. Schlaepfer T. E. (2015). Oxytocin facilitates the extinction of conditioned fear in humans. *Biol. Psychiatry* 78 194–202. 10.1016/j.biopsych.2014.10.015 25542304

[B22] Eichhorn N. Treede R. D. Schuh-Hofer S. (2017). The role of sex in sleep deprivation related changes of nociception and conditioned pain modulation. *Neuroscience* 10.1016/j.neuroscience.2017.09.044 [Epub ahead of print]. 28974374

[B23] Eisenach J. C. Tong C. Curry R. (2015). Phase 1 safety assessment of intrathecal oxytocin. *Anesthesiology* 122 407–413. 10.1097/aln.0000000000000539 25502065 PMC5242190

[B24] Finan P. H. Goodin B. R. Smith M. T. (2013). The association of sleep and pain: an update and a path forward. *J. Pain* 14 1539–1552. 10.1016/j.jpain.2013.08.007 24290442 PMC4046588

[B25] Fujihara H. Serino R. Ueta Y. Sei H. Morita Y. (2003). Six-hour selective REM sleep deprivation increases the expression of the galanin gene in the hypothalamus of rats. *Mol. Brain Res.* 119 152–159. 10.1016/j.molbrainres.2003.09.005 14625082

[B26] Gamal-Eltrabily M. Manzano-Garcia A. (2017). Role of central oxytocin and dopamine systems in nociception and their possible interactions: suggested hypotheses. *Rev. Neurosci.* 27 377–386. 10.1515/revneuro-2017-2068 29222936

[B27] Goldstein-Piekarski A. N. Greer S. M. Saletin J. M. Harvey A. G. Williams L. M. Walker M. P. (2018). Sex, sleep deprivation, and the anxious brain. *J. Cogn. Neurosci.* 30 565–578. 10.1162/jocn_a_01225 29244642 PMC6143348

[B28] Gonzalez-Hernandez A. Manzano-Garcia A. Martinez-Lorenzana G. Tello-Garcia I. A. Carranza M. Aramburo C. (2017). Peripheral oxytocin receptors inhibit the nociceptive input signal to spinal dorsal horn wide-dynamic-range neurons. *Pain* 158 2117–2128. 10.1097/j.pain.0000000000001024 28731982

[B29] Gonzalez-Hernandez A. Rojas-Piloni G. Condes-Lara M. (2014). Oxytocin and analgesia: future trends. *Trends Pharmacol. Sci.* 35 549–551. 10.1016/j.tips.2014.09.004 25270768

[B30] Goodin B. R. Anderson A. J. B. Freeman E. L. Bulls H. W. Robbins M. T. Ness T. J. (2015). Intranasal oxytocin administration is associated with enhanced endogenous pain inhibition and reduced negative mood states. *Clin. J. Pain* 31 757–767. 10.1097/ajp.0000000000000166 25370147 PMC4417654

[B31] Grewen K. M. Light K. C. Mechlin B. Girdler S. S. (2008). Ethnicity is associated with alterations in oxytocin relationships to pain sensitivity in women. *Ethn. Health* 13 219–241. 10.1080/13557850701837310 18568974 PMC4624387

[B32] Grinevich V. Knobloch-Bollmann H. S. Eliava M. Busnelli M. Chini B. (2016). Assembling the puzzle: pathways of oxytocin signaling in the brain. *Biol. Psychiatry* 79 155–164. 26001309 10.1016/j.biopsych.2015.04.013

[B33] Grund T. Goyon S. Li Y. Eliava M. Liu H. Charlet A. (2017). Neuropeptide s activates paraventricular oxytocin neurons to induce anxiolysis. *J. Neurosci.* 37 12214–12225. 10.1523/jneurosci.2161-17.2017 29118105 PMC6596824

[B34] Haack M. Lee E. Cohen D. A. Mullington J. M. (2009). Activation of the prostaglandin system in response to sleep loss in healthy humans: potential mediator of increased spontaneous pain. *Pain* 145 136–141. 10.1016/j.pain.2009.05.029 19560866 PMC2737342

[B35] Heinrichs M. Baumgartner T. Kirschbaum C. Ehlert U. (2003). Social support and oxytocin interact to suppress cortisol and subjective responses to psychosocial stress. *Biol. Psychiatry* 54 1389–1398. 10.1016/s0006-3223(03)00465-467 14675803

[B36] Headache Classification Subcommittee of the International Headache Society (2004). The international classification of headache disorders: 2nd edition. *Cephalalgia* 24(Suppl. 1), 9–160. 14979299 10.1111/j.1468-2982.2003.00824.x

[B37] Kagerbauer S. M. Martin J. Schuster T. Blobner M. Kochs E. F. Landgraf R. (2013). Plasma oxytocin and vasopressin do not predict neuropeptide concentrations in human cerebrospinal fluid. *J. Neuroendocrinol.* 25 668–673. 10.1111/jne.12038 23574490

[B38] Kessner S. Sprenger C. Wrobel N. Wiech K. Bingel U. (2013). Effect of oxytocin on placebo analgesia: a randomized study. *JAMA* 310 1733–1735. 10.1001/jama.2013.277446 24150470

[B39] Kirschbaum C. Kudielka B. M. Gaab J. Schommer N. C. Hellhammer D. H. (1999). Impact of gender, menstrual cycle phase, and oral contraceptives on the activity of the hypothalamus-pituitary-adrenal axis. *Psychosom. Med.* 61 154–162. 10.1097/00006842-199903000-00006 10204967

[B40] Knobloch H. S. Charlet A. Hoffmann L. C. Eliava M. Khrulev S. Cetin A. H. (2012). Evoked axonal oxytocin release in the central amygdala attenuates fear response. *Neuron* 73 553–566. 10.1016/j.neuron.2011.11.030 22325206

[B41] Kragel P. A. Kano M. Van Oudenhove L. Ly H. G. Dupont P. Rubio A. (2018). Generalizable representations of pain, cognitive control, and negative emotion in medial frontal cortex. *Nat. Neurosci.* 21 283–289. 10.1038/s41593-017-0051-57 29292378 PMC5801068

[B42] Kundermann B. Krieg J. C. Schreiber W. Lautenbacher S. (2004a). The effect of sleep deprivation on pain. *Pain Res. Manag.* 9 25–32. 10.1155/2004/949187 15007400

[B43] Kundermann B. Spernal J. Huber M. T. Krieg J. C. Lautenbacher S. (2004b). Sleep deprivation affects thermal pain thresholds but not somatosensory thresholds in healthy volunteers. *Psychosom. Med.* 66 932–937. 10.1097/01.psy.0000145912.24553.c0 15564360

[B44] Lee S. K. Ryu P. D. Lee S. Y. (2013). Differential distributions of neuropeptides in hypothalamic paraventricular nucleus neurons projecting to the rostral ventrolateral medulla in the rat. *Neurosci. Lett.* 556 160–165. 10.1016/j.neulet.2013.09.070 24120435

[B45] Louvel D. Delvaux M. Felez A. Fioramonti J. Bueno L. Lazorthes Y. (1996). Oxytocin increases thresholds of colonic visceral perception in patients with irritable bowel syndrome. *Gut* 39 741–747. 10.1136/gut.39.5.741 9014776 PMC1383401

[B46] Luo L. Becker B. Geng Y. Zhao Z. Gao S. Zhao W. (2017). Sex-dependent neural effect of oxytocin during subliminal processing of negative emotion faces. *Neuroimage* 162 127–137. 10.1016/j.neuroimage.2017.08.079 28877512

[B47] MacDonald K. Feifel D. (2014). Oxytocin’s role in anxiety: a critical appraisal. *Brain Res.* 1580 22–56. 10.1016/j.brainres.2014.01.025 24468203

[B48] Macdonald K. Macdonald T. M. (2010). The peptide that binds: a systematic review of oxytocin and its prosocial effects in humans. *Harv. Rev. Psychiatry* 18 1–21. 10.3109/10673220903523615 20047458

[B49] Macdonald K. S. (2012). Sex, receptors, and attachment: a review of individual factors influencing response to oxytocin. *Front. Neurosci.* 6:194. 10.3389/fnins.2012.00194 23335876 PMC3541513

[B50] Marazziti D. Dell’Osso B. Baroni S. Mungai F. Catena M. Rucci P. (2006). A relationship between oxytocin and anxiety of romantic attachment. *Clin. Pract. Epidemiol. Ment. Health* 2:28. 10.1186/1745-0179-2-28 17034623 PMC1621060

[B51] Martin J. Kagerbauer S. M. Schuster T. Blobner M. Kochs E. F. Landgraf R. (2014). Vasopressin and oxytocin in CSF and plasma of patients with aneurysmal subarachnoid haemorrhage. *Neuropeptides* 48 91–96. 10.1016/j.npep.2013.12.004 24412107

[B52] McQuaid R. J. McInnis O. A. Paric A. Al-Yawer F. Matheson K. Anisman H. (2016). Relations between plasma oxytocin and cortisol: the stress buffering role of social support. *Neurobiol. Stress* 3 52–60. 10.1016/j.ynstr.2016.01.001 27981177 PMC5146198

[B53] Meerlo P. Koehl M. van der Borght K. Turek F. W. (2002). Sleep restriction alters the hypothalamic-pituitary-adrenal response to stress. *J. Neuroendocrinol.* 14 397–402. 10.1046/j.0007-1331.2002.00790.x 12000545

[B54] Menon R. Grund T. Zoicas I. Althammer F. Fiedler D. Biermeier V. (2018). Oxytocin signaling in the lateral septum prevents social fear during lactation. *Curr. Biol.* 28 1066.e6–1078.e6. 10.1016/j.cub.2018.02.044 29551417

[B55] Mutschler I. Ball T. Wankerl J. Strigo I. A. (2012). Pain and emotion in the insular cortex: evidence for functional reorganization in major depression. *Neurosci. Lett.* 520 204–209. 10.1016/j.neulet.2012.03.095 22503725

[B56] Neumann I. D. (2008). Brain oxytocin: a key regulator of emotional and social behaviours in both females and males. *J. Neuroendocrinol.* 20 858–865. 10.1111/j.1365-2826.2008.01726.x 18601710

[B57] Neumann I. D. Slattery D. A. (2016). Oxytocin in general anxiety and social fear: a translational approach. *Biol. Psychiatry* 79 213–221. 10.1016/j.biopsych.2015.06.004 26208744

[B58] Nijs J. Mairesse O. Neu D. Leysen L. Danneels L. Cagnie B. (2018). Sleep disturbances in chronic pain: neurobiology, assessment, and treatment in physical therapist practice. *Phys. Ther.* 98 325–335. 10.1093/ptj/pzy020 29425327

[B59] Paloyelis Y. Krahe C. Maltezos S. Williams S. C. Howard M. A. Fotopoulou A. (2016). The analgesic effect of oxytocin in humans: a double-blind, placebo-controlled cross-over study using laser-evoked potentials. *J. Neuroendocrinol.* 28. 10.1111/jne.12347 26660859 PMC5103211

[B60] Pardo G. V. Goularte J. F. Hoefel A. L. de Castro A. L. Kucharski L. C. da Rosa Araujo A. S. (2016). Effects of sleep restriction during pregnancy on the mother and fetuses in rats. *Physiol. Behav.* 155 66–76. 10.1016/j.physbeh.2015.11.037 26657022

[B61] Patisaul H. B. Scordalakes E. M. Young L. J. Rissman E. F. (2003). Oxytocin, but not oxytocin receptor, is regulated by oestrogen receptor β in the female mouse hypothalamus. *J. Neuroendocrinol.* 15 787–793. 10.1046/j.1365-2826.2003.01061.x 12834440

[B62] Peyron R. Laurent B. Garcia-Larrea L. (2000). Functional imaging of brain responses to pain. A review and meta-analysis (2000). *Neurophysiol. Clin.* 30 263–288. 10.1016/S0987-7053(00)00227-6 11126640

[B63] Pfaff D. Waters E. Khan Q. Zhang X. Numan M. (2011). Minireview: estrogen receptor-initiated mechanisms causal to mammalian reproductive behaviors. *Endocrinology* 152 1209–1217. 10.1210/en.2010-1007 21325045 PMC3060638

[B64] Pires G. N. Bezerra A. G. Tufik S. Andersen M. L. (2016). Effects of acute sleep deprivation on state anxiety levels: a systematic review and meta-analysis. *Sleep Med.* 24 109–118. 10.1016/j.sleep.2016.07.019 27810176

[B65] Rash J. A. Aguirre-Camacho A. Campbell T. S. (2014). Oxytocin and pain: a systematic review and synthesis of findings. *Clin. J. Pain* 30 453–462. 10.1097/AJP.0b013e31829f57df 23887343

[B66] Rash J. A. Campbell T. S. (2014). The effect of intranasal oxytocin administration on acute cold pressor pain: a placebo-controlled, double-blind, within-participants crossover investigation. *Psychosom. Med.* 76 422–429. 10.1097/PSY.0000000000000068 24979580

[B67] Riem M. M. E. Bakermans-Kranenburg M. J. Pieper S. Tops M. Boksem M. A. S. Vermeiren R. R. (2011). Oxytocin modulates amygdala, insula, and inferior frontal gyrus responses to infant crying: a randomized controlled trial. *Biol. Psychiatry* 70 291–297. 10.1016/j.biopsych.2011.02.006 21470595

[B68] Rilling J. K. Demarco A. C. Hackett P. D. Chen X. Gautam P. Stair S. (2014). Sex differences in the neural and behavioral response to intranasal oxytocin and vasopressin during human social interaction. *Psychoneuroendocrinology* 39 237–248. 10.1016/j.psyneuen.2013.09.022 24157401 PMC3842401

[B69] Rolke R. Baron R. Maier C. Tolle T. R. Treede R. D. Beyer A. (2006). Quantitative sensory testing in the german research network on neuropathic pain (DFNS): standardized protocol and reference values. *Pain* 123 231–243. 10.1016/j.pain.2006.01.041 16697110

[B70] Ross H. E. Young L. J. (2009). Oxytocin and the neural mechanisms regulating social cognition and affiliative behavior. *Front. Neuroendocrinol.* 30 534–547. 10.1016/j.yfrne.2009.05.004 19481567 PMC2748133

[B71] Sagaspe P. Sanchez-Ortuno M. Charles A. Taillard J. Valtat C. Bioulac B. (2006). Effects of sleep deprivation on color-word, emotional, and specific stroop interference and on self-reported anxiety. *Brain Cogn.* 60 76–87. 10.1016/j.bandc.2005.10.001 16314019

[B72] Salonia A. Nappi R. E. Pontillo M. Daverio R. Smeraldi A. Briganti A. (2005). Menstrual cycle-related changes in plasma oxytocin are relevant to normal sexual function in healthy women. *Horm. Behav.* 47 164–169. 10.1016/j.yhbeh.2004.10.002 15664019

[B73] Sanford L. D. (2008). Sleep and Pain. *Sleep* 31 753–753. 10.1093/sleep/31.5.753

[B74] Scheele D. Plota J. Stoffel-Wagner B. Maier W. Hurlemann R. (2016). Hormonal contraceptives suppress oxytocin-induced brain reward responses to the partner’s face. *Soc. Cogn. Affect. Neurosci.* 11 767–774. 10.1093/scan/nsv157 26722017 PMC4847696

[B75] Scheele D. Striepens N. Kendrick K. M. Schwering C. Noelle J. Wille A. (2014). Opposing effects of oxytocin on moral judgment in males and females. *Hum. Brain Mapp.* 35 6067–6076. 10.1002/hbm.22605 25094043 PMC6868938

[B76] Schoenen J. Lotstra F. Vierendeels G. Reznik M. Vanderhaeghen J. J. (1985). Substance P, enkephalins, somatostatin, cholecystokinin, oxytocin, and vasopressin in human spinal cord. *Neurology* 35 881–881. 10.1212/wnl.35.6.881 2582309

[B77] Schrimpf M. Liegl G. Boeckle M. Leitner A. Geisler P. Pieh C. (2015). The effect of sleep deprivation on pain perception in healthy subjects: a meta-analysis. *Sleep Med* 16 1313–1320. 10.1016/j.sleep.2015.07.022 26498229

[B78] Schuh-Hofer S. Wodarski R. Pfau D. B. Caspani O. Magerl W. Kennedy J. D. (2013). One night of total sleep deprivation promotes a state of generalized hyperalgesia: a surrogate pain model to study the relationship of insomnia and pain. *Pain* 154 1613–1621. 10.1016/j.pain.2013.04.046 23707287

[B79] Short M. A. Louca M. (2015). Sleep deprivation leads to mood deficits in healthy adolescents. *Sleep Med.* 16 987–993. 10.1016/j.sleep.2015.03.007 26141007

[B80] Silber M. Almkvist O. Larsson B. Stock S. Uvnas-Moberg K. (1987). The effect of oral contraceptive pills on levels of oxytocin in plasma and on cognitive functions. *Contraception* 36 641–650. 10.1016/0010-7824(87)90037-0 3128427

[B81] Singer T. Snozzi R. Bird G. Petrovic P. Silani G. Heinrichs M. (2008). Effects of oxytocin and prosocial behavior on brain responses to direct and vicariously experienced pain. *Emotion* 8 781–791. 10.1037/a0014195 19102589 PMC2672051

[B82] Sivertsen B. Lallukka T. Petrie K. J. Steingrimsdottir O. A. Stubhaug A. Nielsen C. S. (2015). Sleep and pain sensitivity in adults. *Pain* 156 1433–1439. 10.1097/j.pain.0000000000000131 25915149

[B83] Smith M. T. Haythornthwaite J. A. (2004). How do sleep disturbance and chronic pain inter-relate? Insights from the longitudinal and cognitive-behavioral clinical trials literature. *Sleep Med. Rev.* 8 119–132. 10.1016/S1087-0792(03)00044-3 15033151

[B84] Spielberger C. D. Gorsuch R. Lushene R. E. Vagg P. R. (1970). *Manual for the State-Trait Anxiety Inventory.* Palo Alto, CA: Consulting Psychologists Press.

[B85] Striepens N. Kendrick K. M. Maier W. Hurlemann R. (2011). Prosocial effects of oxytocin and clinical evidence for its therapeutic potential. *Front. Neuroendocrinol.* 32 426–450. 10.1016/j.yfrne.2011.07.001 21802441

[B86] Taylor D. J. Mallory L. J. Lichstein K. L. Durrence H. H. Riedel B. W. Bush A. J. (2007). Comorbidity of chronic insomnia with medical problems. *Sleep* 30 213–218. 10.1093/sleep/30.2.213 17326547

[B87] Tracy L. M. Georgiou-Karistianis N. Gibson S. J. Giummarra M. J. (2015). Oxytocin and the modulation of pain experience: implications for chronic pain management. *Neurosci. Biobehav. Rev.* 55 53–67. 10.1016/j.neubiorev.2015.04.013 25956252

[B88] Tracy L. M. Labuschagne I. Georgiou-Karistianis N. Gibson S. J. Giummarra M. J. (2017). Sex-specific effects of intranasal oxytocin on thermal pain perception: a randomised, double-blind, placebo-controlled cross-over study. *Psychoneuroendocrinology* 83 101–110. 10.1016/j.psyneuen.2017.05.028 28601750

[B89] Weisman O. Zagoory-Sharon O. Schneiderman I. Gordon I. Feldman R. (2013). Plasma oxytocin distributions in a large cohort of women and men and their gender-specific associations with anxiety. *Psychoneuroendocrinology* 38 694–701. 10.1016/j.psyneuen.2012.08.011 22999263

[B90] Wilson G. (1988). Wide awake at 3.00 am by choice or by chance? New York, NY: W. H. Freeman and Co.

[B91] Windle R. J. Gamble L. E. Kershaw Y. M. Wood S. A. Lightman S. L. Ingram C. D. (2006). Gonadal steroid modulation of stress-induced hypothalamo-pituitary-adrenal activity and anxiety behavior: role of central oxytocin. *Endocrinology* 147 2423–2431. 10.1210/en.2005-1079 16439458

[B92] Wodarski R. Schuh-Hofer S. Yurek D. A. Wafford K. A. Gilmour G. Treede R. D. (2015). Development and pharmacological characterization of a model of sleep disruption-induced hypersensitivity in the rat. *Eur. J. Pain* 19 554–566. 10.1002/ejp.580 25195796

[B93] Xin Q. Bai B. Liu W. (2017). The analgesic effects of oxytocin in the peripheral and central nervous system. *Neurochem. Int.* 103 57–64. 10.1016/j.neuint.2016.12.021 28065792

[B94] Yang J. Liang J. Y. Li P. Pan Y. J. Qiu P. Y. Zhang J. (2011). Oxytocin in the periaqueductal gray participates in pain modulation in the rat by influencing endogenous opiate peptides. *Peptides* 32 1255–1261. 10.1016/j.peptides.2011.03.007 21439337

[B95] Yarnitsky D. (2010). Conditioned pain modulation (the diffuse noxious inhibitory control-like effect): its relevance for acute and chronic pain states. *Curr. Opin. Anaesthesiol.* 23 611–615. 10.1097/ACO.0b013e32833c348b 20543676

[B96] Yarnitsky D. Bouhassira D. Drewes A. Fillingim R. Granot M. Hansson P. (2015). Recommendations on practice of conditioned pain modulation (CPM) testing. *Eur. J. Pain* 19 805–806. 10.1002/ejp.605 25330039

[B97] Zunhammer M. Geis S. Busch V. Eichhammer P. Greenlee M. W. (2016). Pain modulation by intranasal oxytocin and emotional picture viewing - a randomized double-blind fMRI study. *Sci. Rep.* 6:31606. 10.1038/srep31606 27546446 PMC4992880

[B98] Zunhammer M. Geis S. Busch V. Greenlee M. W. Eichhammer P. (2015). Effects of intranasal oxytocin on thermal pain in healthy men: a randomized functional magnetic resonance imaging study. *Psychosom. Med.* 77 156–166. 10.1097/psy.0000000000000142 25647754

